# Detection and genetic diversity of *Cryptosporidium* in yaks in Xinjiang, China

**DOI:** 10.1016/j.fawpar.2025.e00298

**Published:** 2025-10-28

**Authors:** Zhenjie Zhang, Huigang Zhao, Bowen Zhang, Fuchang Yu, Aiyun Zhao, Junqiang Li, Meng Qi, Rongjun Wang

**Affiliations:** a College of Animal Science and Technology, Tarim University, Alaer 843300, China; b Tarim Animal Disease Diagnosis and Control Engineering, Laboratory of Xinjiang Production and Construction Corps, Alaer 843300, China; c Key Laboratory of Livestock and Forage Resources Utilization around Tarim, Ministry of Agriculture and Rural Affairs, Alaer 843300, China; d College of Veterinary Medicine, Henan Agricultural University, Zhengzhou 450046, China

**Keywords:** *Cryptosporidium*, Infection rate, Species, Genetic diversity, Yak

## Abstract

*Cryptosporidium* spp. is an important protozoan parasite that can cause diarrhea in both humans and animals worldwide. In the present study, a total of 826 yak fecal samples were collected from six counties in Xinjiang and tested for *Cryptosporidium* using PCR. Based on the *SSU* rRNA gene, 20 samples tested positive for *Cryptosporidium*, resulting in an overall infection rate of 2.4 % (20/826). Hejing County exhibited the highest infection rate at 5.6 % (16/288), with significant ``*Cryptosporidium* species and one genotype were identified: *C. bovis* (*n* = 12), *C. parvum* (*n* = 3), *C. ryanae* (n = 3), *C. occultus* (n = 1), and *Cryptosporidium* sp. rat genotype IV (n = 1). Subtyping via the *gp60* gene revealed two subtypes for *C. bovis* (XXVIb, *n* = 4; XXVIc, n = 4), one subtype for *C. ryanae* (XXIa, n = 1), and one subtype for *C. parvum* (IIdA19G1, n = 1). Phylogenetic analysis indicated that these subtypes clustered with reference sequences from other regions and hosts, without distinct geographical or host specific isolation. In conclusion, the prevalence of *Cryptosporidium* infection in yaks in Xinjiang is low, and the subtypes of *Cryptosporidium* exhibit genetic diversity among different bovine species.

## Highlights

• Low *Cryptosporidium* infection rate (2.4 %) in Xinjiang yaks, with significant regional variation.
• Four species and one genotype were identified in yaks.
• Potential cross-species transmission suggested by shared *Cryptosporidium* subtypes.
• No distinct geographical isolation from other regions and hosts.

## 1 Introduction

*Cryptosporidium* spp. is a globally prevalent protozoan pathogen that affects both humans and animals (Ryan et al., 2021). To date, at least 48 confirmed *Cryptosporidium* species and over 120 genotypes have been reported (Ryan et al., 2021; Tůmová et al., 2023). Among these, 10 species and one genotype have been identified in yaks, including *Cryptosporidium bovis*, *C. ryanae*, *C. parvum*, *C. andersoni*, *C. occultus*, *C. ubiquitum*, *C. hominis*, *C. canis*, *C. xiaoi*, *C. struthionis*, and *Cryptosporidium* yak genotype (Wang Lan et al., 2018; Ma et al., 2014; Qi et al., 2015).

The yak (*Bos grunniens*), an ancient cold-resistant bovid species, holds significant economic importance, with over 95 % of the global yak population residing in China. In this country, yaks primarily inhabit high altitude regions (3000–5000 m) across the Xizang Autonomous Region, Qinghai Province, Xinjiang Uygur Autonomous Region, and parts of autonomous prefectures or counties in Gansu and Sichuan provinces, serving as a vital source of meat, milk, and hides (Lan Lan et al., 2018). Previous studies have reported an overall *Cryptosporidium* infection rate of 10.5 % (1192/8012) in Chinese yaks from 2001 to 2021, with higher prevalence observed during the cold season and among yaks under 12 months of age (Taghipour et al., 2020; Geng et al., 2021). Although *Cryptosporidium* infections in yaks have been investigated in Xizang, Qinghai, Gansu, and Sichuan provinces, limited research has focused on the prevalence of this parasite in yaks from Xinjiang (Li et al., 2016; Wang et al., 2019).

Existing studies on *Cryptosporidium* in yaks have primarily concentrated on the provinces of Qinghai, Xizang, and Gansu, leaving the extensive pastoral regions of Xinjiang underrepresented (Geng et al., 2021; Li et al., 2016; Li et al., 2020; Ma et al., 2014; Mi et al., 2014). As China's largest province, Xinjiang possesses unique ecological zones and distinct yak husbandry practices; however, it remains insufficiently studied in terms of Cryptosporidium transmission dynamics. Consequently, the present study seeks to characterize the prevalence, species, and genotypes of *Cryptosporidium* in yaks from Xinjiang, northwestern China.

## 2 Materials and methods

### 2.1 Feces sample collection

**Fig. 1:**
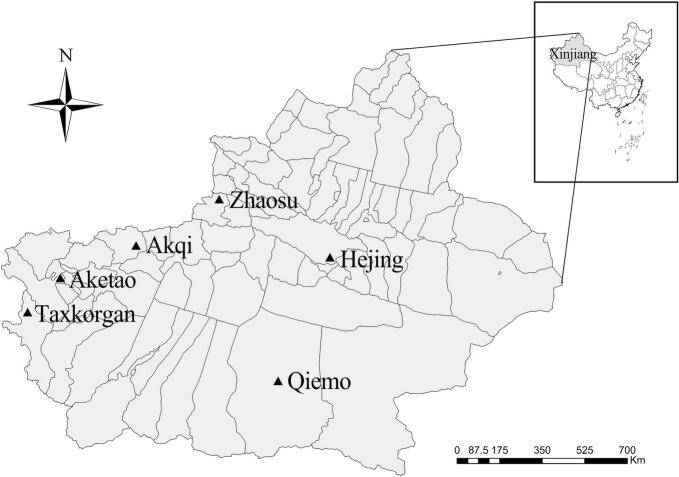
Geographical distribution of fecal samples collected from yaks in Xinjiang, northwestern China.

From April 2022 to November 2023, a total of 826 fresh fecal samples were randomly collected from free ranging yaks across six counties in Xinjiang (Fig. 1). Fresh feces were collected from the ground immediately after defecation was observed. To prevent cross-contamination, freshly excreted fecal samples were meticulously selected from the ground during collection. Using disposable sterile gloves, only the middle portion of the feces, which had not come into contact with the ground was collected. Gloves were replaced between each sample collection. Following collection, samples were individually placed in clean centrifuge tubes and stored in a sealed manner.

Each fecal sample, weighing approximately 20 g was placed into a labeled plastic bag, promptly transported to the laboratory and stored at 4 °C for subsequent analysis. The age of the sampled yaks could not be accurately recorded, as they were raised in an extensive free-ranging system where individuals of all age groups cohabited.

### 2.2 DNA extraction

Approximately 200 mg of the stool sample was transferred to a 2 mL centrifuge tube for DNA extraction using the commercial E.Z.N.A.TM Stool DNA Kit (Omega Bio-Tek Inc., Norcross, GA, USA), in accordance with the manufacturer's protocol. The extracted DNA was subsequently stored at −20 °C for future analysis.

### 2.3 PCR amplification

The presence of *Cryptosporidium* in yak fecal samples was detected using polymerase chain reaction (PCR) targeting the small subunit ribosomal RNA (*SSU* rRNA) gene. Positive samples identified as *C. parvum*, *C. bovis*, and *C. ryanae* were further analyzed through PCR targeting the 60 kDa glycoprotein (*gp60*) gene. The primer sequences, annealing conditions, and amplicon sizes are detailed in Table 1. Each 25 μL PCR reaction mixture comprised 1 μL of DNA template, 12.5 μL of 2× Easy Taq PCR Super Mix (+dye), 0.3 μL of forward primer, 0.3 μL of reverse primer, and 10.9 μL of double-distilled water (ddH_2_O).

**Table 1:** The primers sequence, annealing and amplicon size in the present study.

Gene	Sequence (5′-3′)	Annealing (°C)	Size (bp)	Reference
*SSU* rRNA	18SiCF1: GAC ATA TCA TTC AAG TTT CTG ACC	58	763	Alves et al., 2003
	18SiCR1: CTG AAG GAG TAA GGA ACA ACC			
	18SiCF2: CCT ATC AGC TTT AGA CGG TAG G	58	587	
	18SiCR2: TCT AAG AAT TTC ACC TCT GAC TG			
Bovis *gp*60	Bovis-*gp60*-F1: ATG CGA CTT ACG CTC TAC ATT ACT CT	55		Wang et al., 2021
	Bovis-*gp60*-R1: GAC AAA ATG AAG GCT GAG ATA GAT GGG A			
	Bovis-*gp60*-F2: CCT CTC GGC ATT TAT TGC CCT	55	1300	
	Bovis-*gp60*-R2: ATA CCT AAG GCC AAA TGC TGA TGA A			
AL *gp*60	AL3531: ATA GTC TCC GCT GTA TTC	50	1280	Alves et al., 2003
	AL3535: GGA AGG AAC GAT GTA TCT			
	AL3532: TCC GCT GTA TTC TCA GCC	55	850	
	AL3534: GCA GAG GAA CCA GCA TC			
Ry *gp*60	Ry-*gp60*-F1: GCT CGA GTT CTG AGT CGA	55	1068	Yang et al., 2020
	Ry-*gp60*-R1: ATA CCG TTA AAA TGA AGG CCA A			
	Ry-*gp60*-F2: CCT CAG ATA ATG AGC AGT CTA	55	1024	
	Ry-*gp60*-R2: GAT GGG ATA ACA TAT CTA TAA CCA AA			

### 2.4 Sequencing and phylogenetic analysis

Positive PCR amplicons were subjected to Sanger sequencing by Xinjiang Youkang Biotechnology Co., Ltd. The resultant sequences underwent initial quality checks were conducted and alignment using ChromasPro software (http://www.technelysium.com.au/ChromasPro.html). Subsequently, homology comparisons against the GenBank database utilizing the Basic Local Alignment Search Tool (BLAST; https://blast.ncbi.nlm.nih.gov/Blast.cgi) to ascertain species and genotype identities. Multiple sequence alignment and visual corrections of the sequences were then performed using Clustal X (version 2.1; http://www.clustal.org/).

To elucidate the genetic relationships among various subtypes, the obtained *gp60* sequences were subjected to phylogenetic analysis alongside reference sequences. Specifically, the aligned sequences were analyzed employing the Maximum Likelihood (ML) method implemented in MEGA 11 software (http://www.megasoftware.net/previousVersions.php). The reliability of the phylogenetic trees was assessed through non-parametric bootstrap resampling with 1000 replicates, based on a nucleotide substitution model.

### 2.5 Statistical analysis

All statistical analyses were performed using IBM SPSS Statistics software (www.ibm.com/products/spssstatistics). The infection rates of *Cryptosporidium* were calculated, and 95 % confidence intervals (CIs) were determined. Statistical significance was defined as a two-tailed *P*-value of less than 0.05.

## 3 Results

### 3.1 Infection rate of *Cryptosporidium* spp. in yaks

Based on the *SSU* rRNA gene analysis, a total of 20 samples tested positive for *Cryptosporidium*, resulting in an overall infection rate of 2.4 % (20/826) in yaks. Among the six counties sampled, Hejing County exhibited the highest infection rate at 5.6 % (16/288), followed by Qiemo County at 2.0 % (2/100), Akqi County at (1.0 %, 1/100), and Zhaosu County at 0.5 % (1/216). No infections were detected in Aketao County or the Tajik Autonomous County of Taxkorgan (hereafter referred to as Taxkorgan) (see Table 2). Significant differences in *Cryptosporidium* infection rates were observed among the six sampling sites (χ^2^ = 6.708, *P* < 0.01). (See Table 3.)

**Table 2:** Infection rate and species/subtypes of *Cryptosporidium* in yaks in Xinjiang.

Location	No. Positives/ No. Samples (%)	95 % CI	*Cryptosporidium* spp./Subtype (n)				
			*C. bovis*	*C. parvum*	*C. ryanae*	*C. occultus*	*Cryptosporidium* rat genotype IV
Hejing County	16/288 (5.6)	2.9–8.2	10/XXVIb (4), XXVIc (2)	2/−	2/−	1/−	1/−
Qiemo County	2/100 (2.0)	0.8–4.8		1/IIdA19G1 (1)	1/XXIa (1)	/	/
Zhaosu County	1/216 (0.5)	0.4–1.4	1/XXVIc (1)	/	/	/	/
Akqi County	1/100 (1.0)	1.0–3.0	1/XXVIc (1)	/	/	/	/
Aketao County	0/22 (0)	/	/	/	/	/	/
Taxkorgan County	0/100 (0)	/	/	/	/	/	/
Total	20/826 (2.4)	1.4–3.5	12/XXVIb (4), XXVIc (4)	3/IIdA19G1 (1)	3/XXIa (1)	1	1

Note: - nontypable at *gp60* gene.

**Table 3:** The prevalence and molecular characterization of *Cryptosporidium* in yaks in China from 2013 to 2024.

**Location**	**Infection rate of *Cryptosporidium***	**Species and genotype**^a^	**Reference**
Qinghai	24.2 % (142/586)	*C. bovis* (*n* = 31), *C. parvum* (*n* = 16), *C. ryanae* (n = 5), ND (n = 87)^b^, *C. bovis* + *C. parvum* (*n* = 2), *C. bovis* + *C. ryanae* (*n* = 1)	Mi et al., 2014
Qinghai	30.0 % (98/327)	*C. bovis* (*n* = 56), *C. ryanae* (*n* = 33), *C. andersoni* (n = 2), *C. ubiquitum* (n = 1), *C. xiaoi* (n = 1), *Cryptosporidium* yak type (*n* = 2), *C. bovis* + *C. ryanae* (n = 3)	Ma et al., 2014
Gansu	6.0 % (7/117)	*C. bovis* (n = 2), *C. ryanae* (n = 2), *C. parvum* (n = 2), *C. ubiquitum* (n = 1)	Qi et al., 2015
Xizang	9.1 % (4/44)	*C. parvum* (*n* = 4)	
Sichuan	1.2 % (1/84)	*C. parvum* (n = 1)	
Qinghai	3.7 % (11/300)	*C. bovis* (n = 4), *C. parvum* (*n* = 6), *C. ryanae* (n = 1)	
Qinghai	28.5 % (158/554)	*C. andersoni* (*n* = 72), *C. bovis* (*n* = 47), *C. occultus* (n = 2), *Cryptosporidium ryanae* cattle type (*n* = 35), *Cryptosporidium ryanae* buffalo type (n = 2)	Li et al., 2016
Qinghai	11.3 % (39/344)	*C. bovis* (*n* = 11), *C. andersoni* (n = 5), *C. struthionis* (n = 5), *C. ryanae* (n = 6), *C. hominis* (n = 4), *C. parvum* (n = 5), *C. canis* (n = 3)	Lan et al., 2018
Xizang	1.4 % (8/577)	*C. bovis* (n = 1), *C. andersoni* (*n* = 7)	Wu et al., 2019
	1.3 % (2/150)	*C. bovis* (n = 2)	Li et al., 2020
	33.0 % (30/91)	*C. bovis* (n = 5), *C. parvum* (n = 2)^c^,	Peng et al., 2024
Total	15.8 % (500/3174)	*C. bovis* (*n* = 159), *C. ryanae* (*n* = 47), *C. parvum* (*n* = 36), *C. andersoni* (*n* = 86), *C. occultus* (n = 2), *C. ubiquitum* (n = 2), *C. hominis* (n = 4), *C. canis* (n = 3), *C.xiaoi* (n = 1), *C. struthionis* (n = 5), *C. bovis* + *C. parvum* (n = 2), *C. bovis* + *C. ryanae* (n = 4), *Cryptosporidium* yak genotype (n = 2), *Cryptosporidium* ryanae cattle type (n = 35), *Cryptosporidium* ryanae buffalo type (n = 2), ND (*n* = 87)	

a : The data in this table are the results of PCR testing of yak fecal samples from 2013 to 2024.

b : ND indicates that the species/genotype is not determined.

c : 7 of the 30 positive samples were successfully sequenced and identified to the species.

### 3.2 Species of *Cryptosporidium* in yaks

All *Cryptosporidium*-positive samples were successfully sequenced for the *SSU* rRNA gene. Sequence alignment analysis revealed the presence of four distinct *Cryptosporidium* species along with the *Cryptosporidium* sp. rat genotype IV. Among these, *C. bovis* was the most prevalent species with a total of 12 occurrences, followed by *C. parvum* (*n* = 3), *C. ryanae* (n = 3), *C. occultus* (*n* = 1), and *Cryptosporidium* sp. rat genotype IV (n = 1) (see Table 2). Notably, all four *Cryptosporidium* species and *Cryptosporidium* sp. rat genotype IV were detected in Hejing County. In Qiemo County, only *C. parvum* and *C. ryanae* were identified; whereas in Zhaosu County and Akqi County, only *C. bovis* was detected (refer to Table 2).

### 3.3 Subtyping of ***C. parvum***, ***C. bovis*** and C. ryanae in yaks

At the *gp60* gene locus, only one out of three *C. parvum* isolates was successfully subtyped, yielding the IIdA19G1 subtype (*n* = 1, Table 2). This subtype exhibited 100 % sequence homology with the previously reported *C. parvum* subtype IIdA19G1 (accession no. MF074760) found in dairy cattle from China.

Among the twelve *C. bovis* samples, eight were successfully subtyped, identifying two genotypes (XXVIb, *n* = 4; XXVIc, n = 4) and five distinct sequence types. One sequence exhibited 99.83 % homology with *C. bovis* subtype XXVIb (MZ977192) from yaks in China, with nucleotide substitutions at positions 171 (A → G) and 347 (C → A). Another sequence showed 99.74 % homology with the same reference subtype (MZ977192), featuring substitutions at positions 55 (C → T), 162 (A → G), and 338 (C → A). Two sequences displayed 100 % homology with *C. bovis* subtype XXVIb (MZ977192). One sequence presented 99.66 % homology with *C. bovis* subtype XXVIc (PP157571) from dairy cattle in China, with substitutions at positions 76 (T → C), 539 (C → T), 830 (A → T), and 1104 (A → G). Additionally, three sequences showed 100 % homology with *C. bovis* subtype XXVIc (MZ977158) from yaks in China.

For *C. ryanae*, one out of three samples was successfully subtyped as XXIa (n = 1), sharing 99.25 % sequence homology with *C. ryanae* subtype XXIa (OP925775) from dairy cattle in China, with nucleotide substitutions at positions 7 (G → A) and 59 (G → C).

### 3.4 The phylogenetic analysis of subtypes in ***C. parvum***, ***C. bovis*** and *C. ryanae*

**Fig. 2:**
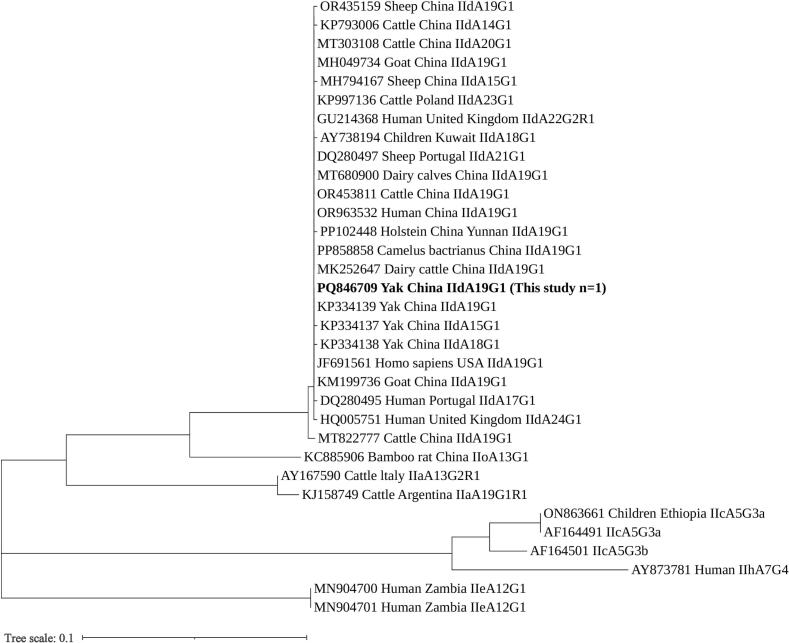
Phylogenetic relationships among representative sequences of the *Cryptosporidium parvum* 60 kDa glycoprotein (*gp60*) gene obtained from Xinjiang, analyzedusing the maximum likelihood method based on the General Time Reversible model. Bootstrap values exceeding 50 % from 1000 pseudo-replicates are displayed. The isolate identified in this study is highlighted in bold text.

The *C. parvum* IIdA19G1 subtype examined in this study clustered with sequences from the IId subtype family derived from yaks in Qinghai (Mi et al., 2014; Li et al., 2016), dairy cattle in Guangdong (Feng et al., 2019), cattle in Poland (Kaupke and Rzeżutka, 2015), and humans in the UK (Pangasa et al., 2023), forming a cohesive clade. Within this clade, sequences from various hosts (yaks, dairy cattle, humans) and geographical origins (China, Europe) intermingled without forming host-specific or geographically distinct subclusters (Fig. 2).

**Fig. 3:**
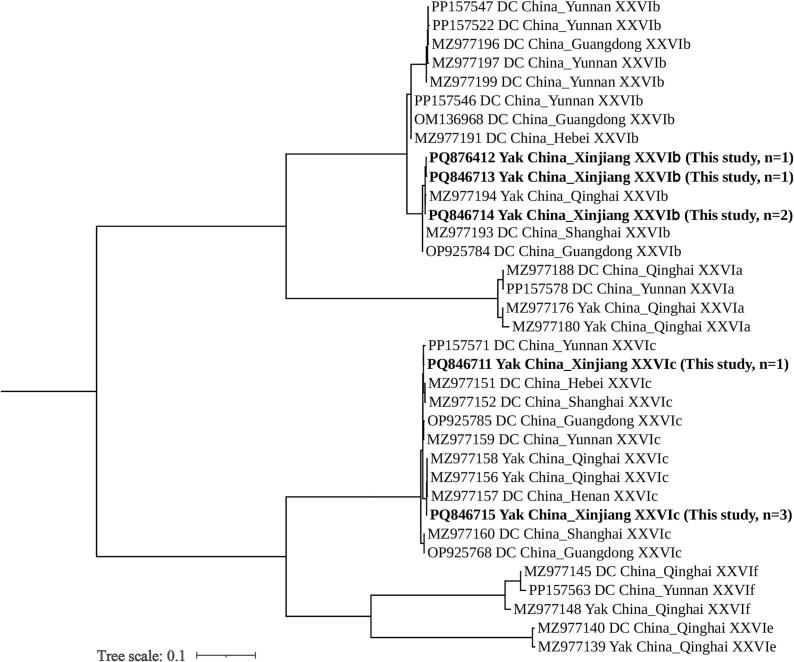
Phylogenetic relationships among representative sequences of the *Cryptosporidium bovis* 60 kDa glycoprotein (*gp60*) gene obtained from Xinjiang, employing the maximum likelihood method based on the General Time Reversible model. Bootstrap values exceeding 50 % from 1000 pseudo-replicates are displayed. The isolate identified in this study is highlighted in bold text.

For *C. bovis*, the XXVIb subtype clustered with the XXVIb reference sequence from dairy cattle in Guangdong but formed an independent subcluster characterized by minor nucleotide variations, suggesting potential intrasubtype differentiation driven by host factors (yak vs. dairy cattle). The XXVIc subtype exhibited a high degree of clustering with XXVIc sequences from yaks in Qinghai and dairy cattle in Hebei, with some sequences demonstrating 100 % homology (Fig. 3).

**Fig. 4:**
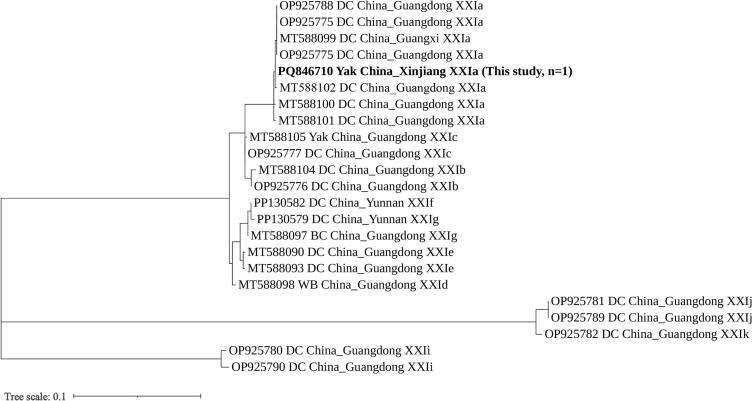
Phylogenetic relationships among representative sequences of the *Cryptosporidium ryanae* 60 kDa glycoprotein (*gp60*) gene obtained from Xinjiang, employing the maximum likelihood method based on the General Time Reversible model. Bootstrap values exceeding 50 % from 1000 pseudo-replicates are displayed. The isolate identified in this study is highlighted in bold text.

The *C. ryanae* XXIa subtype in this study clustered with the XXIa reference sequence from dairy cattle in Guangdong, forming a distinct branch separate from other subtypes (e.g., XXIb, XXIc) with only minor genetic evolutionary differences. This branch included both dairy cattle and yak hosts, confirming the ability of the XXIa subtype to cross infect Bovidae animals (Fig. 4). Notably, sequences from Xinjiang and Guangdong did not exhibit geographical isolation despite spatial distance, indicating a lack of significant geographical differentiation.

## 4 Discussion

The present study elucidats the infection status and molecular characteristics of *Cryptosporidium* in yaks within this region through the analysis of 826 fecal samples collected from six counties in Xinjiang. The overall infection rate of *Cryptosporidium* in yaks in Xinjiang was determined to be 2.4 % (20/826), which is lower than the rates observed in other major yak-producing areas of China, such as Qinghai (30.0 %) and Gansu (6.0 %) (Ma et al., 2014; Qi et al., 2015). This rate is only higher than that reported in Sichuan (1.2 %) (Qi et al., 2015) and Xizang (1.3 %–1.4 %) (Wu et al., 2019; Li et al., 2020). This discrepancy may be partially attributed to the extensive communal grazing systems of the sampled population, which limited the ability to conduct age-stratified analysis of infection patterns. Host age is a well-established determinant of *Cryptosporidium* susceptibility, with immature immune function in juveniles typically leading to higher prevalence in younger cohorts (Ryan et al., 2021; Zhang et al., 2021). The absence of age metadata thus represents a significant limitation in this study. In terms of spatial distribution, Hejing County exhibited the highest infection rate (5.6 %) and was the only area where all five *Cryptosporidium* species were identified. Conversely, no positive samples were detected in Akto County and Taxkorgan County, indicating notable regional disparities in infection rates.

The present study identified four species of *Cryptosporidium* and one genotype. The dominant species was *C. bovis* (12/20), followed by *C. parvum* (3/20) and *C. ryanae* (3/20), with fewer instances of *C. occultus* (1/20) and *Cryptosporidium* rat genotype IV (1/20). Among these, *C. bovis*, recognized as the most prevalent *Cryptosporidium* species in yaks, aligns with findings from numerous domestic studies. Its high detection rate may be attributed to the physiological characteristics of yaks and the adaptability of this species to Bovidae animals (Ma et al., 2014; Li et al., 2016; Wang et al., 2018). *C. parvum* exhibits zoonotic potential, with three cases identified in this study, indicating a public health risk. *C. occultus*, primarily found in cattle and rodents, is rare in yaks, with only two cases reported in Qinghai (Li et al., 2016). This detection further substantiates its capability for cross-host infection. *Cryptosporidium* rat genotype IV has previously been reported exclusively in rodents (Kváč et al., 2018; Zhang et al., 2021). However, in this study, we detected *Cryptosporidium* rat genotype IV in yak feces. Although stringent sampling measures were implemented to minimize contamination risk, the natural infection status of this genotype in yaks requires further verification due to its host specificity.

Through *gp60* genotyping, the subtype characteristics of the three main species were elucidated. Only one isolate of *C. parvum* was successfully genotyped as IIdA19G1, which exhibited 100 % homology with the sequence of dairy cattle-derived IIdA19G1 (MF074760) in China. This subtype has been previously confirmed to possess zoonotic potential in bovids (Ryan et al., 2021). However, as only one positive sample was identified in this study, the prevalence of this subtype in yak populations and its actual risk to human health remain uncertain. Among *C. bovis*, eight isolates were successfully genotyped, including two subtypes, XXVIb and XXVIc, with five sequence variants identified. Both XXVIb and XXVIc have been reported in dairy and beef cattle, indicating potential cross-infection among Bovidae species (Deng et al., 2024). One isolate of *C. ryanae* was successfully genotyped as XXIa, marking the first instance of this subtype being identified in yaks (previously predominantly found in dairy cattle), suggesting the adaptive capacity of the XXIa subtype to infect yaks (Yang et al., 2020).

In comparative studies with domestic research, the *C. parvum* subtype IIdA19G1, previously identified in intensively and semi-intensively farmed cattle herds in Qinghai, Gansu, and Inner Mongolia, has now been detected in extensively free-ranging yaks in Xinjiang (Ma et al., 2014; Li et al., 2016; Wang et al., 2018). This finding suggests that the host adaptation range of this subtype has expanded beyond intensive cattle systems. Furthermore, subtypes XXVIb and XXVIc of *C. bovis*, as well as XXIa of *C. ryanae*, which have been rarely reported in Chinese yaks, are newly recorded in Xinjiang, thereby extending their known geographical distribution into the arid and semi-arid pastoral regions of China. From a public health perspective, the IIdA19G1 subtype, recognized as a zoonotic variant associated with human cases in rural China, where contact with livestock is common, positions Xinjiang yaks as an unrecognized reservoir.

## 5 Conclusions

The infection rate of *Cryptosporidium* in yaks in Xinjiang is relatively low; however, there exists a rich diversity of species and subtypes, which are closely genetically related to *Cryptosporidium* found in Bovidae from other regions of China. This information serves as foundational data for the prevention and control of yak cryptosporidiosis.

## CRediT authorship contribution statement

**Zhenjie Zhang:** Writing – original draft, Validation, Investigation, Formal analysis. **Huigang Zhao:** Supervision, Investigation, Data curation. **Bowen Zhang:** Validation, Supervision, Software. **Fuchang Yu:** Methodology, Investigation, Formal analysis. **Aiyun Zhao:** Data curation, Conceptualization. **Junqiang Li:** Writing – review & editing, Supervision, Project administration. **Meng Qi:** Writing – review & editing, Supervision, Project administration, Conceptualization. **Rongjun Wang:** Writing – review & editing, Supervision, Project administration, Funding acquisition, Conceptualization.

## Declaration of competing interest

The authors declare that they have no competing interests.
